# Noninvasive in vivo deoxycytidine kinase (dCK)-PET identifies tumor-draining lymph nodes upon immune checkpoint inhibitor therapy

**DOI:** 10.1038/s44303-025-00133-8

**Published:** 2026-01-06

**Authors:** Cécile Philippe, Jonathan Cotton, Gregory D. Bowden, Simone Pöschel, Philipp Knopf, Barbara Schörg, Irene Gonzalez-Menendez, Dominik Sonanini, Lukas Flatz, Martin Allen-Auerbach, Caius G. Radu, Johannes Czernin, Leticia Quintanilla-Martinez, Marcus Hacker, Bernd J. Pichler, Andreas Maurer, Manfred Kneilling

**Affiliations:** 1 Division of Nuclear Medicine, Department of Biomedical Imaging and Image-guided Therapy, Medical University of Vienna, Vienna, Austria; 2 Werner Siemens Imaging Center, Department of Preclinical Imaging and Radiopharmacy, Eberhard Karls University, Tuebingen, Germany; 3 Cluster of Excellence iFIT (EXC 2180) “Image-Guided and Functionally Instructed Tumor Therapies”, Eberhard Karls University, Tuebingen, Germany; 4 Core Facility Flow Cytometry of the Medical Faculty Tübingen, Eberhard Karls University, Tuebingen, Germany; 5 Institute of Pathology and Neuropathology and Comprehensive Cancer Center Tuebingen, Eberhard Karls University, Tübingen, Germany; 6 Department of Medical Oncology and Pneumology, Eberhard Karls University, Tuebingen, Germany; 7 Department of Dermatology, Eberhard Karls University, Tuebingen, Germany; 8 Ahmanson Translational Theranostics Division, Department of Molecular and Medical Pharmacology, UCLA, Los Angeles, California USA; 9 Department of Molecular and Medical Pharmacology, UCLA, Los Angeles, CA USA

**Keywords:** Imaging the immune system, Cancer, Molecular imaging, Radionuclide imaging

## Abstract

Efficient application of immunotherapy necessitates advanced whole-body imaging techniques to monitor sites of immune cell activation. Deoxycytidine kinase (dCK), a key enzyme in the deoxynucleotide salvage pathway, is upregulated in proliferating immune cells and can be targeted by the radiotracers [^18^F]FAC (preclinical) and [^18^F]CFA (clinical), allowing for noninvasive monitoring of immune activation in lymphatic organs via positron emission tomography (PET). In this study, we aimed to assess the efficacy of [^18^F]FAC in detecting immune activation upon immune checkpoint inhibitor therapy (CIT). In vitro, activated T cells and macrophages exhibited significantly higher [^18^F]FAC uptake compared to their naïve counterparts. In vivo, preclinical [^18^F]FAC-PET/MRI revealed a CIT-induced significant increase in [^18^F]FAC uptake in tumor-draining lymph nodes (TDLNs) compared to contralateral lymph nodes, independent of tumor responsiveness. This phenomenon was absent in TDLNs of sham-treated experimental mice. Ex vivo cell sorting further confirmed elevated [^18^F]FAC uptake in T cells from TDLNs following CIT. Consistently, [^18^F]CFA-PET/CT imaging in metastatic melanoma patients demonstrated CIT-induced enhanced regional LN uptake. Together, these findings establish a strong correlation between CIT-induced immune activation and [^18^F]FAC/[^18^F]CFA uptake, underscoring the critical role of TDLNs in cancer immuotherapy. The radiotracers [^18^F]FAC and [^18^F]CFA provide valuable tools for noninvasive monitoring of immune cell activation, potentially unveiling tumor-microenvironment-related resistance mechanisms and advancing the utility of PET imaging in immunotherapy monitoring and patient stratification.

## Introduction

Immune checkpoint inhibitor therapies (CITs) have revolutionized the treatment of many cancers. For example, the inhibition of immune checkpoints such as programmed cell death protein 1 (PD-1) and its ligand (PD-L1)^1^ has resulted in exceptional clinical success among patients diagnosed with late-stage metastatic melanoma^2^, non-small cell lung cancer^3,4^, renal cancer^5^, and colorectal cancer^6^. Despite this remarkable efficacy, the majority of cancer patients are poor, partial, or nonresponders to CIT^7–9^. As the mechanisms of resistance are largely unknown, there is an unmet need to predict and monitor therapeutic responses. Early detection of treatment failure would enable us to modify the treatment protocol and help us avoid immune-related adverse events (colitis, neurotoxicity, cardiotoxicity)^10^, in addition to reducing the costs to the healthcare system. However, due to the complexity of cancer biology, including the composition of the mostly heterogeneous tumor microenvironment (TME), predicting CIT outcomes is highly challenging. Currently, patient stratification is based on histopathology and immunohistochemical analyses of tumor biopsies, which do not provide dynamic information or reflect tumor heterogeneity^11^. Since immune cell activation in lymphatic organs is essential for generating an effective immune response, the TME and lymphoid organs are of interest during CIT^12^. Mounting evidence underscores the critical role of the tumor-draining lymph node (TDLN) for effective systemic anti-tumor immune responses^13–18^. TDLNs serve as key immunological hubs, fueling systemic tumor-specific T cell activity that enhances the efficacy of cancer immunotherapy. Upon activation, CD4⁺ and CD8⁺ T cells migrate from the LNs into the TME, where they initiate anti-tumor immune responses. Moreover, as demonstrated in a preclinical study^13^, CIT-induced immune cell activation predominantly occurs within TDLNs and is associated with the accumulation of CD8^+^ T cells. Disruption of this process - either through surgical resection of TDLNs or chemical blockade of T cell migration - abrogates CIT efficacy, and thus underscores the critical role of the presence of the TDLNs in mediating effective immune responses. Consequently, routine removal of TDLNs for staging or therapeutic purposes may need to be reconsidered, given its potential to compromise immune function and long-term treatment success. A deeper understanding of how tumors interact with TDLNs is crucial for optimization of CIT strategies and to improve the patients’ outcomes. Advances in molecular pathology and immune profiling of TDLNs may ultimately enable more precise immunotherapy selection and better prediction of treatment efficacy in the era of personalized medicine.

Molecular imaging techniques such as positron emission tomography (PET) offer a noninvasive alternative for disease monitoring and the prediction of immunotherapeutic response^19–24^. In this context, several radiotracers have been developed to target distinct pathways within the immune system^21^. For instance, immuno-PET agents targeting immune checkpoints, such as [^89^Zr]DFO-atezolizumab (which binds to PD-L1), or T cell-specific tracers like [⁸⁹Zr]Df-IAB22M2C (which binds to CD8), have demonstrated clinical potential in tracking immune responses^25,26^. Beyond targeting cell surface markers, imaging metabolic alterations within immune cells provides a complementary strategy to assess immune activation. Hence, in addition to its widespread use in oncological imaging, the radiolabeled glucose analog 2-deoxy-2-[^18^F]fluoro-D-glucose ([^18^F]FDG) is also applied to determine enhanced glycolysis in activated immune cells^27^. Targeting specific metabolic pathways, such as the nucleotide salvage pathway, a metabolic process upregulated in highly proliferating immune cells, enables a more precise identification of immune cell activation upon CIT^28,29^. Thus, substrates of the deoxyribonucleotide salvage pathway, such as 3’-deoxy-3’-[^18^F]fluorothymidine ([^18^F]FLT)^30,31^, [^18^F]fluoro-arabinosyl guanine ([^18^F]F-AraG)^32–34^, 2-chloro-2’-deoxy-2’-[^18^F]fluoro-9-β-D-arabinofuranosyl-adenine ([^18^F]CFA)^35^ and its functional analog for murine studies 1-(2’-deoxy-2’-[^18^F]fluoro-β-D-arabinofuranosyl)-cytosine ([^18^F]FAC)^36^, can be used to monitor immune cell activation. Previous studies have shown that [^18^F]FLT provides a readout of thymidine kinase 1 activity, which correlates with cell proliferation^30^. [^18^F]FLT has been used to map cell proliferation in the spleen following CIT^31^. Similarly, [^18^F]F-AraG, a substrate of deoxyguanosine kinase (dGK), has demonstrated the ability to image activated T cells, serving as an early predictor of immune response to CIT^32^. Increased [^18^F]F-AraG uptake in the TDLNs clearly indicated responsiveness to anti-PD-1 therapy. Furthermore, enhancement of [^18^F]F-AraG bone marrow uptake represents a promising predictive imaging biomarker for patient stratification and treatment guidance^34^. In parallel, deoxycytidine kinase (dCK) activity - another critical axis for nucleotide-based imaging - can be monitored with [^18^F]FAC and [^18^F]CFA. [^18^F]FAC has demonstrated sensitivity to identify localized immune cell activation within the spleen and TDLNs in an experimental oncoretrovirus tumor model^36^. Additionally, [^18^F]FAC-PET has been shown to selectively image liver-infiltrating activated T cells in a model of autoimmune hepatitis^37^ and brain-infiltrating leukocytes (predominantly CD11b^+^ myeloid cells and CD4^+^ T cells) in a multiple sclerosis model^38^. Unlike its murine analog [^18^F]FAC, [^18^F]CFA contains a purine rather than a pyrimidine base, making it less susceptible to deamination. This structural difference enhances its stability in humans^35,39^. A first-in-human study validated [^18^F]CFA as a highly specific PET tracer for dCK, showing probe accumulation in tissues with high dCK expression, such as bone marrow and secondary lymphoid organs^39^. The tracer was also studied in gliobastoma patients undergoing CIT, where enhanced [^18^F]CFA accumulation was observed in both tumor and secondary lymphoid organs post-therapy^40^. Building on these previous investigations, we aimed to evaluate the efficacy of dCK-PET as a tool for detecting systemic immune activation following CIT. To date, the potential of dCK-PET for monitoring CIT has been rarely explored. Using well-established tumor mouse models - both CIT-sensitive and -resistant - we aimed to assess potential CIT-induced immune cell activation in primary and secondary lymphatic organs using [^18^F]FAC. This was complemented by in vitro cell uptake studies and extensive ex vivo analyses, including biodistribution, radioactive cell sorting, histopathology, immunohistochemistry (IHC), and flow cytometry. Finally, we conducted PET imaging with [^18^F]FAC’s clinical analog, [^18^F]CFA, in metastatic melanoma patients before and after CIT.

## Results

### Preclinical in vivo [^18^F]FAC-PET/MRI reveals CIT-mediated [^18^F]FAC uptake in TDLNs

**Fig. 1:**
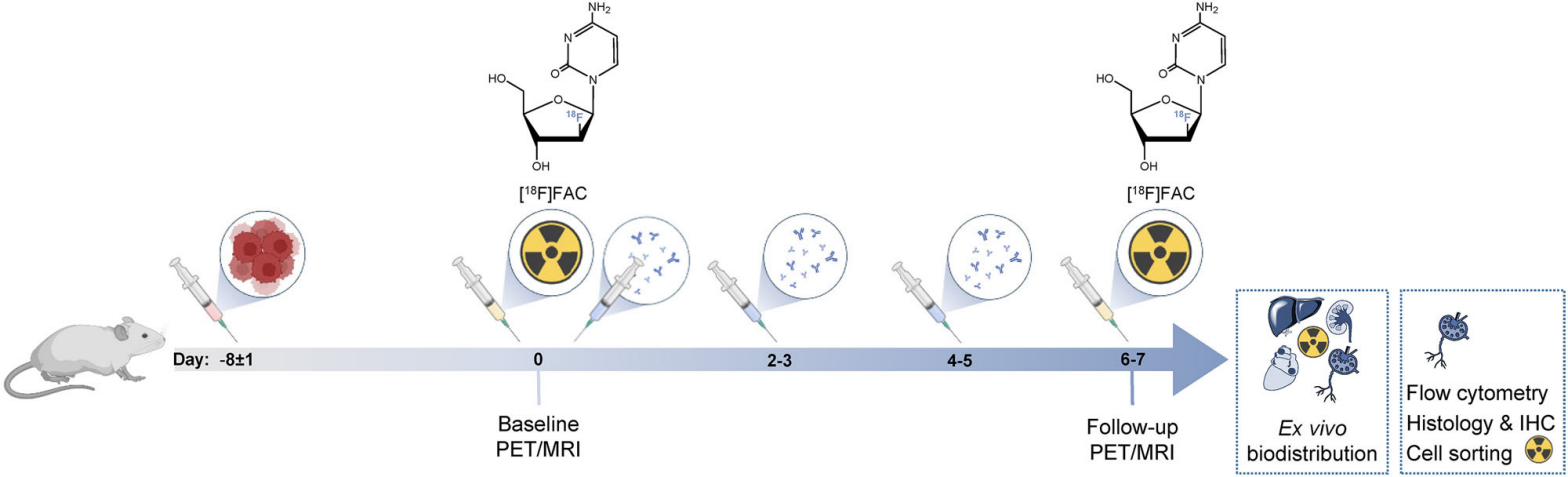
Preclinical experimental set-up. Timeline of the in vivo experiments: tumor cell inoculation, treatment (anti-PD-L1 mAb or isotype mAb), [^18^F]FAC-PET/MRI and ex vivo analyses.

We selected two syngeneic CIT-sensitive (MC38, CT26)^13,41–44^ or CIT-resistant (B16-F10, 4T1)^41,45–47^ tumor models on a C57BL/6 J (MC38, B16-F10) or BALB/c (CT26, 4T1) background (Table 1). Baseline and follow-up PET/magnetic resonance imaging (MRI) were performed on anti-PD-L1 or isotype mAb-treated mice (Fig. 1). Determination of the tumor volume confirmed the CIT responsiveness or treatment failure of these models (Fig. S1).

**Table 1:** Overview of the different exogenous tumor mouse models

Mouse strain:	C57BL/6 J	BALB/c
CIT-sensitive tumors:	**MC38** (colon adenocarcinoma)^13,41,42^	**CT26** (colon adenocarcinoma)^13,41–44^
CIT-resistant tumors:	**B16-F10** (melanoma)^41,45,46^	**4T1** (breast cancer)^41,47^

The bold values indicate the specific cell lines.

**Fig. 2:**
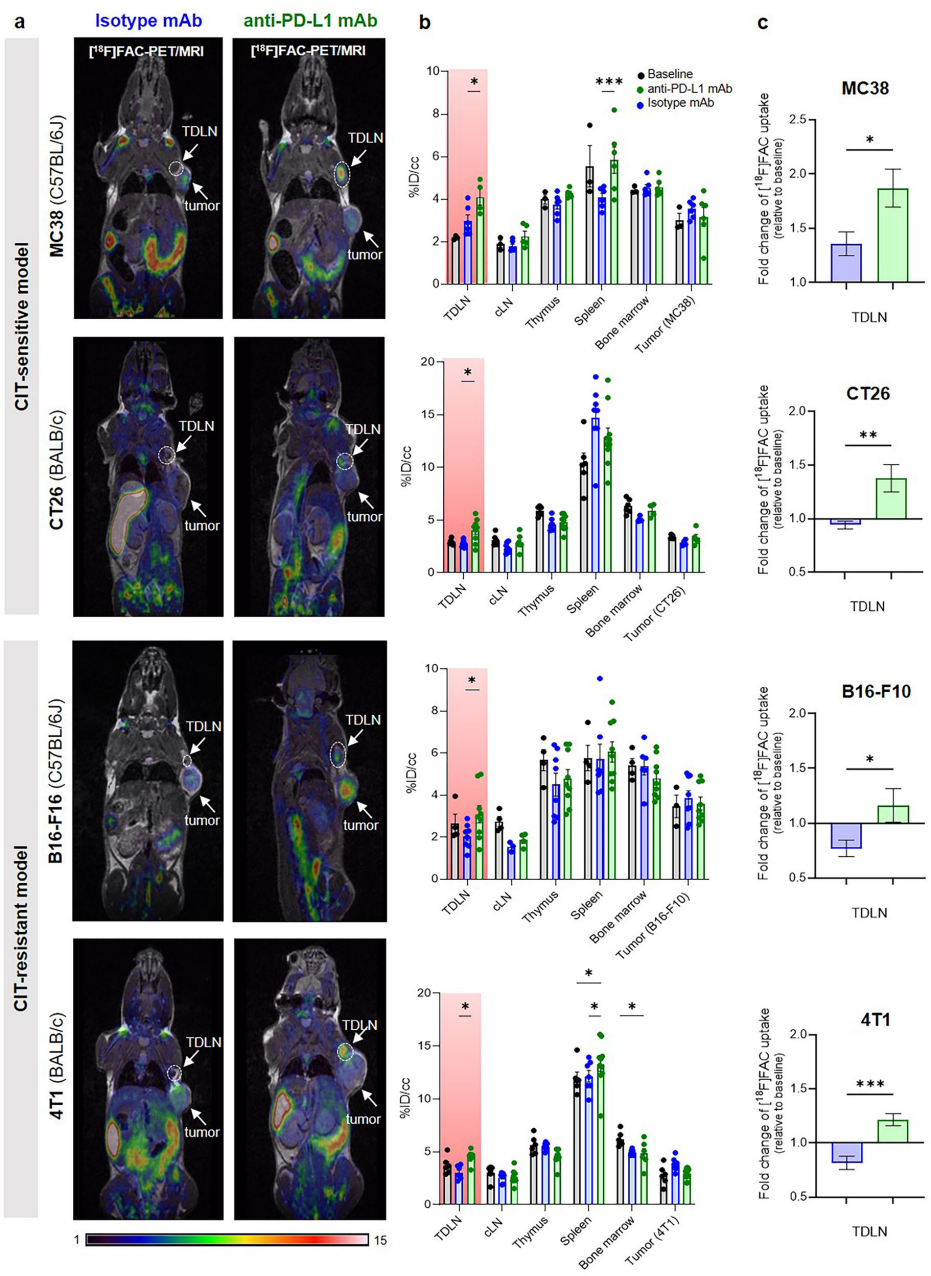
Preclinical in vivo imaging of [^18^F]FAC. **a** Representative [^18^F]FAC-PET/MR scans (coronal) of the examined tumor models. **b** [^18^F]FAC in vivo uptake quantification in primary and secondary lymphoid organs as well as tumors (*n* = 4–10 animals per group). **c** Fold change of [^18^F]FAC uptake in the TDLN compared to baseline. The data are expressed as means ± SEM (*p < 0.05). %ID/cc = percentage injected dose per cubic centimeter, *TDLN* tumor-draining lymph node, *cLN* control (contralateral) lymph node.

Across all four exogenous tumor models, [^18^F]FAC uptake in the TDLNs (brachial or axillary) was significantly higher in mice treated with anti-PD-L1 mAbs compared to isotype mAb controls, irrespective of tumor sensitivity to CIT (anti-PD-L1 mAb groups: 3–4.5%ID/cc, isotype mAb control group: 2–3%ID/cc; Fig. 2a–c). Among all analyzed organs, the spleen exhibited the highest [^18^F]FAC uptake (6–14%ID/cc), followed by the primary lymphatic organs bone marrow (6–7%ID/cc) and thymus (4–6%ID/cc) (Fig. 2b). Spleen uptake was significantly increased in MC38 and 4T1 models following anti-PD-L1 mAb treatment compared to isotype controls. Additionally, [^18^F]FAC accumulated in tumors across all models, with no differences in uptake between baseline and follow-up scan or between treatment regimens (Fig. 2b).

Ex vivo biodistribution analysis confirmed the in vivo [^18^F]FAC-PET/MRI results; however, only a trend toward increased [^18^F]FAC uptake in TDLNs following CIT was observed (Fig. S2). Notably, BALB/c mice exhibited higher spleen uptake than C57BL/6J mice. Moreover, significant intestinal [^18^F]FAC uptake was observed in both strains (Fig. S2), consistent with the findings of Brewer et al.^48^, who demonstrated that intestinal epithelial cells actively take up [^18^F]FAC, likely due to their high proliferation rate. Additionally, the large number of immune cells residing in the intestinal mucosa may further contribute to the observed [^18^F]FAC accumulation^48^.

To exclude that [^18^F]FAC uptake in the femur results from defluorination, we assessed the tracer’s in vitro and ex vivo serum stability. <0.2% fluorine-18 was detectable in our in vitro experiments for up to 240 min and ex vivo for over 60 min (Fig. S3).

### Ex vivo and in vitro analyses of [^18^F]FAC uptake confirm its uptake by proliferating cells

**Fig. 3:**
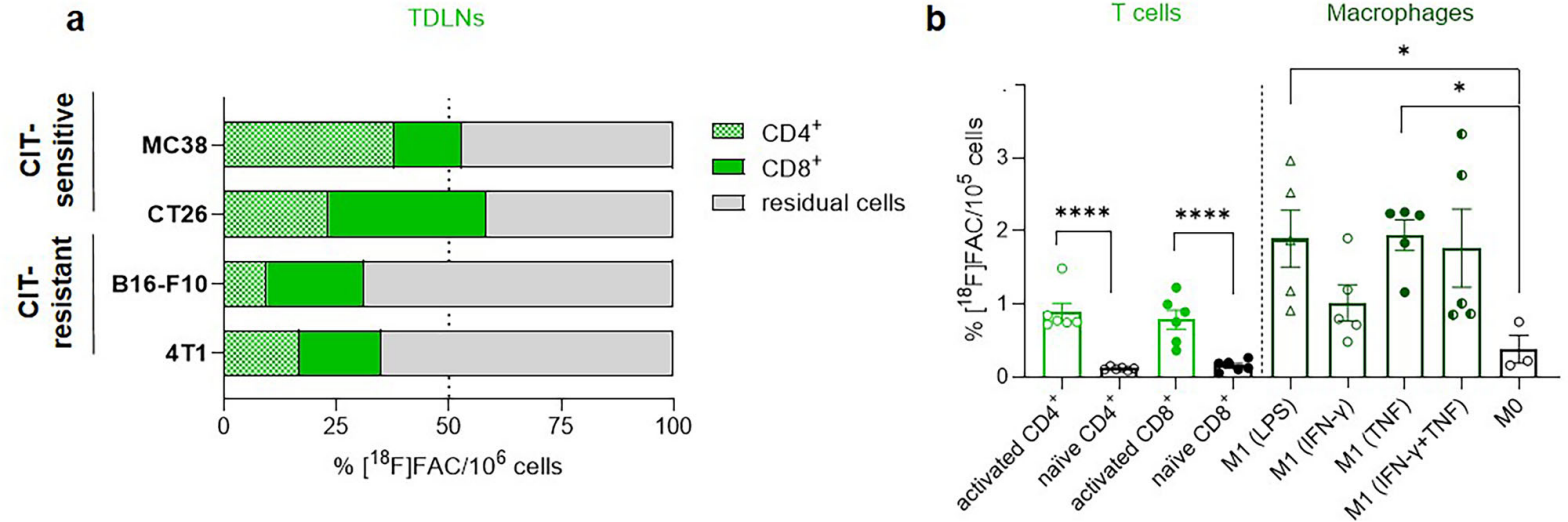
Ex vivo and in vitro cell uptake of [^18^F]FAC. **a** Ex vivo uptake in T cells (CD4^+^ and CD8^+^) and non T cells (CD4^-^ and CD8^-^) from the tumor-draining lymph nodes (TDLNs) of mice treated with the anti-PD-L1 mAb, following 80 min of [^18^F]FAC distribution under anesthesia (*n* = 1, consisting of 3 pooled lymph nodes per tumor model). **b** In vitro [^18^F]FAC uptake in T cells and macrophages after 60 min of incubation. The data are expressed as means ± SEM (*p < 0.05, ****p < 0.0001) (*n* = 4–5 individual experiments, each in duplicate; the uptake results from T cells and macrophages originating from C57BL/6J and BALB/c mice were combined).

Subsequent to the imaging, we performed ex vivo analyses to assess [^18^F]FAC-associated radioactivity in CD4^+^ and CD8^+^ T cells isolated from the TDLNs of anti-PD-L1 mAb-treated mice bearing CIT-sensitive or CIT-resistant tumors. T cells from CIT-sensitive models (MC38, CT26) exhibited predominant (>50%) [^18^F]FAC uptake, whereas lower uptake was detected in the nonsensitive models (B16-F10: ~31%, 4T1: ~35%) (Fig. 3a). Building on these ex vivo findings, we extended our investigation to in vitro studies to further elucidate [^18^F]FAC uptake dynamics in immune cells. Radu et al.^26^ extensively characterized [^18^F]FAC uptake in naïve and activated thymocytes and splenocytes. Building on this work, we specifically focused on immune cells that may be activated during CIT, including T cells and macrophages. We evaluated [^18^F]FAC uptake in naïve and activated CD4⁺ and CD8⁺ T cells, as well as in naïve macrophages (M0) and those stimulated (M1) for innate (LPS) or adaptive (IFN-γ, TNF, IFN-γ & TNF) responses derived from C57BL/6J (Th1-prone) and BALB/c (Th2-prone) mice. No significant differences in [^18^F]FAC uptake were observed between mouse strains in either T cell or macrophage (Fig. S4a, b), allowing data combination. [^18^F]FAC uptake steadily increased over time (30–120 min) in activated T cells and LPS-stimulated macrophages, while uptake in IFN-γ- and TNF-stimulated macrophages plateaued after 60 min (Fig. S4c, d). Our results demonstrated significantly higher [^18^F]FAC uptake in activated T cells and stimulated macrophages compared to their naïve counterparts (Fig. 3b). In detail, activated CD4^+^ and CD8^+^ T cells exhibited a 10-fold increase in [^18^F]FAC uptake relative to naïve T cells. Similarly, M1 macrophages, stimulated for innate or adaptive immune responses, showed a 5-fold increase compared to unstimulated M0 macrophages; with IFN-γ stimulation alone resulting in a more modest 2–3-fold rise. [^18^F]FAC uptake in naïve T cells and unstimulated macrophages was negligible, comparable to unspecific uptake levels. Notably, M1-stimulated macrophages exhibited approximately 2-fold higher [^18^F]FAC uptake than activated T cells.

Besides immune cells, we additionally investigated [^18^F]FAC uptake in the tumor cell lines used in our experimental mouse models. All tumor lines, except B16-F10, demonstrated increasing uptake over time (Fig. S4e). The uptake in CT26 tumor cells was similar to that observed in stimulated macrophages (~2% [^18^F]FAC/10^5^ cells), while the other tumor cell lines exhibited significantly higher uptake (~4–9% [^18^F]FAC/10^5^ cells), up to 5-fold greater than activated T cells, or up to 2-fold higher than in stimulated macrophages (~2% [^18^F]FAC/10^5^ cells) (Fig. S4f). Interestingly, in vitro [^18^F]FAC uptake was higher in tumor cells than in immune cells, contrasting with the findings from in vivo PET studies.

### Ex vivo analysis of the effects of CIT on TDLNs and control LNs in CIT-sensitive and CIT-resistant tumor models

**Fig. 4:**
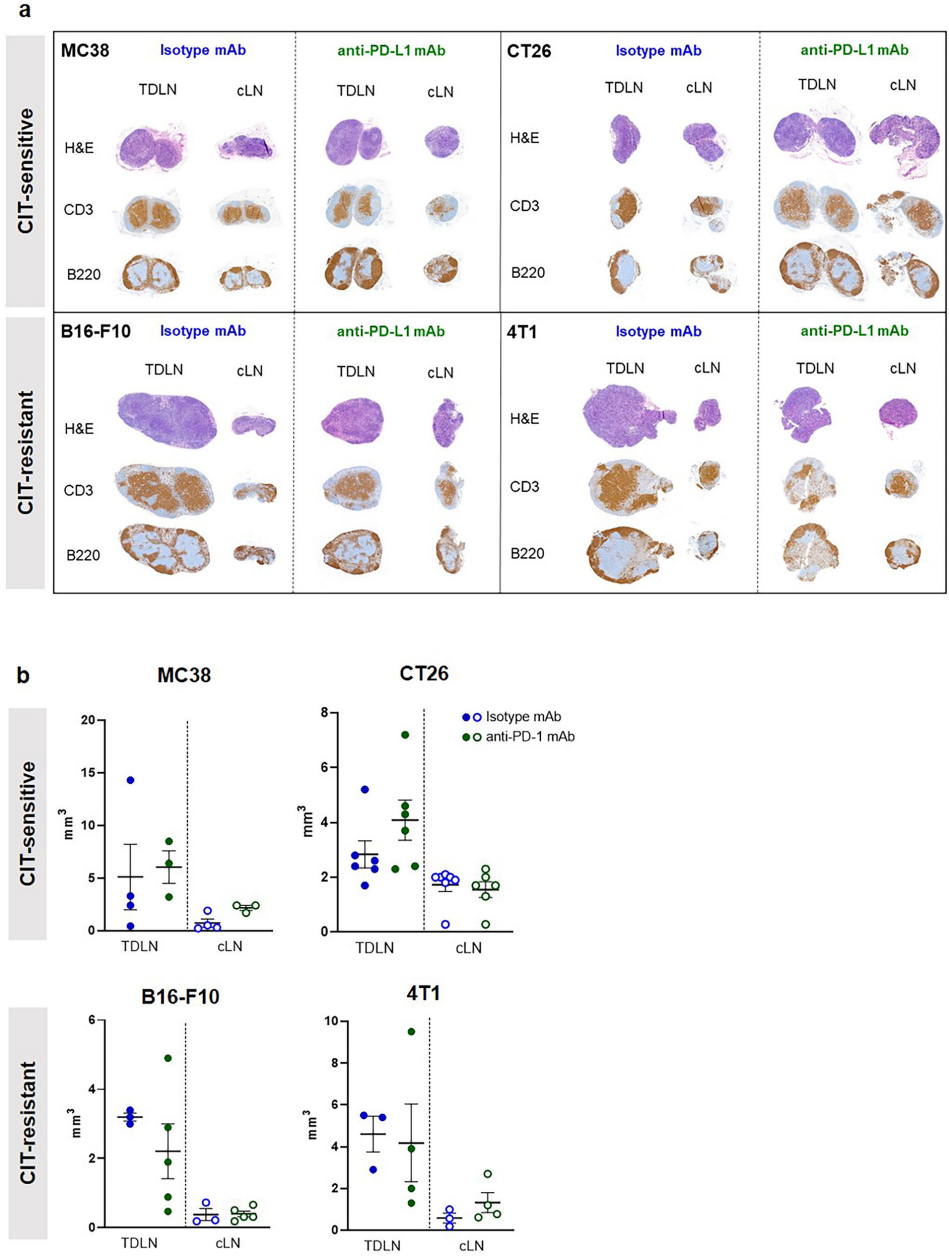
Ex vivo and in vivo analyses of lymph nodes. **a** Histology and IHC staining of representative lymph nodes. H&E = hematoxylin and eosin, CD3 ≙ T cells, B220 ≙ B cells. **b** MRI-based volumes of TDLNs and cLNs (n = 3–6 lymph nodes per group). *TDLN* tumor-draining lymph node, *cLN* control (contralateral) lymph node.

To correlate the prominent [^18^F]FAC uptake in TDLNs with cell subtypes, we performed histopathological (H&E) and IHC analyses using CD3 staining for T cells and B220 staining for B cells, on TDLNs and contralateral control LNs (cLNs) from all experimental groups. H&E staining confirmed the proper architecture of TDLNs and cLNs. The cross-sections of the TDLNs were larger than those of their cLNs, independent of anti-PD-L1 or isotype mAb treatment (Fig. 4a). Consistently, MRI-based determination of TDLN volumes revealed no significant differences between treatment groups, indicating that volume alone did not account for the enhanced [^18^F]FAC uptake observed in vivo exclusively in the anti-PD-L1 mAb treatment group (Fig. 4b). Qualitative IHC analysis revealed a slight decrease in the T cells (CD3^+^) and a moderate increase in the B cells (B220^+^) within the TDLN of anti-PD-L1-treated MC38- and 4T1-bearing mice. No changes were observed in B16-F10-bearing mice between treatments. Furthermore, fewer T cells and slightly more B cells were detected in the TDLNs of CT26-bearing mice treated with the isotype mAb compared to those treated with the PD-L1 mAb (Fig. 4a).

We conducted quantitative multicolor flow cytometry of TDLNs and cLNs, concentrating on T and myeloid cells for further correlation of our [^18^F]FAC-PET results (Fig. S5). No significant differences were found in the percentage of macrophages (F4/80^+^), T cells (CD3^+^), and activated T cells (CD4^+^ CD25^+^, CD4^+^ CD69^+^, CD8^+^ CD69^+^) between anti-PD-L1 and isotype mAb treatments, as well as between TDLNs and cLNs. Interestingly, CD11b^+^ myeloid cells significantly increased after CIT in MC38- and 4T1-bearing mice, with a similar trend in CT26-bearing mice, while no changes were observed in B16-F10-bearing mice. Additionally, cLNs tended to have more T cells and CD11b^+^ cells than TDLNs. CD4^+^/CD8^+^ ratios remained consistent across tumor models and treatment groups.

### Clinical [^18^F]CFA-PET investigations reveal CIT-induced immune cell activation in TDLNs

**Fig. 5:**
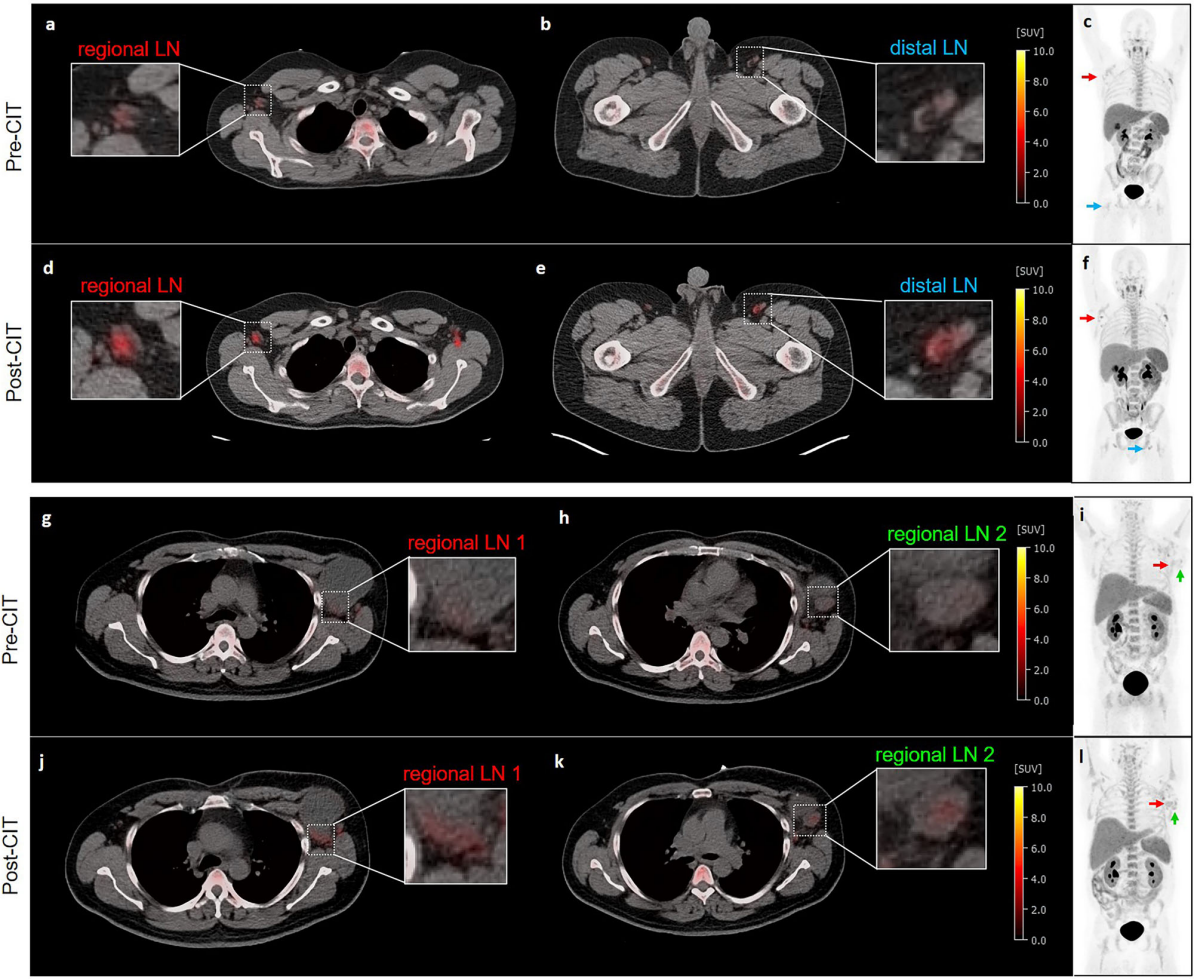
[^18^F]CFA-PET/CT in metastatic melanoma patients before and after CIT. 27-year-old male with subcentimeter lung lesions: baseline axial images showing (**a**) regional and (**b**) distal lymph node (LN) uptake of [^18^F]CFA. **c** Baseline maximum intensity projection image (MIP). **d** Axial, (**e**) distal LN uptake and **f** MIP after immune checkpoint inhibitor therapy (CIT) (red arrow: regional LN, blue arrow: distal LN). 47-year-old male patient with advanced melanoma and left axillary lymph node metastases: baseline axial images showing (**g**, **h**) regional LN uptake of [^18^F]CFA. **i** Baseline MIP. **j**, **k** Axial LN uptake and **l** MIP after immune checkpoint inhibitor therapy (CIT) (red arrow: regional LN 1, green arrow: regional LN 2).

Two metastatic melanoma patients underwent [^18^F]CFA-PET/computed tomography (CT) before and 2–4 weeks after the onset of combined CIT (anti-PD1 mAb + anti-CTLA4 mAb). In a 27-year-old male with small (subcentimeter) lung lesions, [^18^F]CFA uptake increased by 86% in an axillary lymph node in regional proximity to the metastatic lesions after CIT. In addition, in the follow-up scan, we detected enhanced [^18^F]CFA uptake (118%) in a distal inguinal lymph node (Table 2, Fig. 5a–f). A 47-year-old male with advanced melanoma and metastases in the left axillary lymph nodes showed 34% and 111% increases in regional lymph nodes (Table 2, Fig. 5g–l). These findings suggest CIT-induced immune activation within lymph nodes close to suspected metastatic melanoma lesions, presumably TDLNs, and within one distant lymph node.

**Table 2:** Lymph node size and [^18^F]CFA uptake before and after CIT in melanoma patients.

Patient	Lymph node	Pre-CIT		Post-CIT		
		Size [mm]	SUVmax [g/mL]	Size [mm]	SUVmax [g/mL]	SUVmax increase [%]
27-year-old man (Fig. 5a–f)	Regional	20 × 14	2.8	17 × 11	5.2	85.7
	Distal	17 × 8	2.2	20 × 10	4.8	118
47-year-old man (Fig. 5g–l)	Regional 1	44 × 31	2.6	38 × 26	3.5	34
	Regional 2	26 × 21	1.7	27 × 19	3.6	111

## Discussion

Our [^18^F]FAC-PET/MRI data confirmed the tracer’s characteristic distribution in the primary and secondary lymphatic organs, as well as in the intestines, aligning with prior findings by Radu et al.^36^. In most organs, [^18^F]FAC uptake showed no differences between baseline, anti-PD-L1, and isotype control mAb treatments. Similarly, tumor uptake did not differ between CIT-treated and sham-treated (isotype mAb) experimental groups, nor between the CIT-sensitive and CIT-resistant cohorts. The most prominent CIT-related effect was observed in the TDLNs, where [^18^F]FAC uptake significantly increased in the anti-PD-L1 mAb-treated group. Notably, this effect was not restricted to CIT-sensitive models (MC38 and CT26) but was also present in CIT-resistant models (B16-F10 and 4T1), suggesting immune cell activation even in mice with nonresponding tumors. The slightly increased tracer uptake in the TDLN of the MC38 model under isotype control conditions likely reflects a certain degree of basal immune activation within the TDLN, which can occur due to tumor-associated inflammation. This effect appears to be tumor model–dependent, as it was not observed in the other models, and may relate to differences in the inherent immunogenicity and TME between these tumor types. Ex vivo analysis of TDLNs from CIT groups confirmed [^18^F]FAC accumulation within T cells, with a more pronounced uptake in CIT-sensitive models compared to resistant ones. Furthermore, in our clinical study of melanoma patients, we identified enhanced [^18^F]CFA uptake in the lymph nodes near the metastatic lesions and in one distant lymph node as a result of CIT. Together, our findings highlight the capacity dCK-targeting tracers to detect immune cell activation within TDLNs, independent of tumor responsiveness. This distinguishes [^18^F]FAC from the dGK-targeted tracer [^18^F]F-AraG, whose uptake in the TDLNs was enhanced exclusively upon tumor responsiveness to CIT^32^. Moreover, [^18^F]F-AraG tumor uptake was also increased following CIT, offering more specific insights into T cell-mediated responses associated with effective immunotherapy^32^. In contrast, [^18^F]FAC provides a broader assessment of immune activation within TDLNs, regardless of tumor responsiveness.

In vitro experiments revealed significant differences in [^18^F]FAC uptake among the four experimental tumor cell lines cell. However, these variances were not observed in our in vivo [^18^F]FAC-PET/MRI data, likely due to tumor-specific differences of the TME. Focusing on [^18^F]FAC uptake by naïve and activated immune cells, we confirmed significantly enhanced uptake in activated T cells. Additionally, we detected significantly increased uptake in activated macrophages, suggesting that [^18^F]FAC can serve as a marker for immune activation across multiple cell types. Notably, in vivo [¹⁸F]FAC uptake by tumors was relatively low, while uptake in lymphoid organs was high—opposite to the in vitro pattern. In vitro, tumor cells are exposed to an overabundance of nutrients and freely available tracer, unopposed by competing nucleosides, resulting in a high uptake. In contrast, in vivo, tracer accumulation in tumors is limited by multiple factors, including CDA-mediated metabolism, competition for nucleoside transporters, delivery barriers, and context-dependent regulation of dCK activity^35,36^. The higher uptake in lymphoid organs reflects the preferential accumulation of [¹⁸F]FAC in metabolically active immune cells.

No differences in [^18^F]FAC uptake were observed between leukocytes originating from the two experimental mouse strains (C57BL/6J and BALB/c). In contrast, the in vivo distribution of [^18^F]FAC differed between the two strains, as BALB/c mice (CT26 and 4T1 tumor models) exhibited significantly greater accumulation in the spleen than C57BL/6J mice (MC38 and B16-F10 tumor models). This is likely due to the increased density of splenic T cells in BALB/c mice, as well as increased cytokine production by their splenocytes^49^.

The notable [^18^F]FAC uptake observed in the TDLNs following CIT was not related to increased lymph node size, as the TDLNs of the sham-treated groups exhibited comparable volumes. Furthermore, the elevated tracer accumulation did not correlate with a higher abundance of activated immune cells in the TDLNs. Flow cytometry revealed a moderately reduced T cells presence in the TDLNs, particularly in those treated with anti-PD-L1 mAb, except in the B16-F10 group. IHC analyses of TDLNs from CIT-treated MC38 and 4T1 tumor-bearing mice corroborated these findings. Moreover, we observed no differences in the abundance of activated T cells between the CIT and sham-treated control groups within the identified tumor models. Interestingly, CD11b⁺ myeloid cells significantly increased after CIT in MC38- and 4T1-bearing mice, with a similar trend in CT26-bearing mice, whereas no changes were observed in B16-F10-bearing mice. We hypothesize that this myeloid activation is mediated by reinvigorated T cells releasing IFN-γ and chemokines, which recruit and activate myeloid populations, consistent with previous studies^50^. Conversely, B16-F10 melanomas are considered immunologically ‘cold,’ characterized by low T cell infiltration, impaired IFN signaling, and tumor-intrinsic β-catenin–driven exclusion of dendritic cells, which may prevent the initiation of such immune activation cascades^51^.

As IHC and flow cytometry could not fully substantiate our imaging findings, these results suggest that [^18^F]FAC-PET may detect metabolic alterations associated with immune activation that are not discernible through conventional immunophenotyping techniques. A possible explanation is that the metabolic activation detected by dCK-PET imaging most likely precedes measurable changes in immune cell composition or surface activation markers. Indeed, previous studies have demonstrated that dCK expression is upregulated upon T cell activation and that tracer uptake in lymphoid tissues correlates with dCK mRNA levels^35,36^. Accordingly, dCK-PET reflects early metabolic reprogramming associated with lymphocyte activation—such as enhanced nucleoside salvage and DNA precursor turnover—occurring prior to detectable proliferation or shifts in T cell and macrophage subsets. Consistent with this interpretation, our ex vivo analysis confirmed [¹⁸F]FAC accumulation in both CD4⁺ and CD8⁺ T cells with the TDLNs, indicating that the signal originates from activated lymphocyte populations. This positions [^18^F]FAC-PET as a potentially more sensitive modality for monitoring immune responses within TDLNs, offering insights beyond those provided by flow cytometry or IHC. However, the potential of dCK-PET must ultimately be assessed compared with imaging with the gold standard of PET, [^18^F]FDG.

We previously described that metabolic changes following CIT could already be identified via [^18^F]FDG-PET in primary and secondary lymphoid organs^52^. Our study revealed that [^18^F]FDG-PET enabled discrimination between CIT responders and nonresponders based on enhanced [^18^F]FDG uptake in the spleen (preclinical study) and bone marrow (retrospective analysis of cancer patients) of CIT responders. Additionally, Seith et al.^53^ described an effective systemic immune response in patients undergoing CIT, evidenced by significantly increased spleen activity on [^18^F]FDG-PET two weeks after treatment initiation. In another clinical study by Ribas et al.^31^, the uptake of the cell proliferation tracer [^18^F]FLT in the spleen was significantly increased in patients with metastatic melanoma upon immunotherapy with the CTLA4-blocking antibody tremelimumab, regardless of therapy response. However, this study did not report significant changes in [^18^F]FDG uptake in the spleen before and after treatment. Further supporting the role of the TDLN in CIT, Alsaid et al.^54^ demonstrated increased uptake of [^89^Zr]IAB42M1-14, an anti-CD8^+^ minibody, in the TDLNs of tumor-bearing mice during CIT. Collectively, these studies underscore the potential of immuno-PET as a valuable tool for detecting metabolic changes in lymphoid organs in response to CIT. Our data further support the essential role of TDLNs in promoting anticancer immune responses and indicate the potential harmful effects of standard-of-care removal. By employing [^18^F]FAC- and [^18^F]CFA-PET, we demonstrate a novel, noninvasive strategy to visualize CIT-induced immune cell activation and to distinguish TDLNs in both CIT-sensitive and CIT-resistant tumors. These findings provide deeper insights into the involvement and interplay of the deoxyribonucleoside salvage pathway in antitumor immunity and suggest that dCK-PET imaging could serve as a translational tool for identifying mechanisms of systemic immune activation.

## Methods

### Cell lines and tumor models

Dulbecco’s modified Eagle’s medium (DMEM) and RPMI-1640 medium were purchased from Sigma Aldrich (St. Louis, MO, USA) and supplemented with 10% fetal calf serum (FCS), 100 U/mL penicillin, and 100 µg/mL streptomycin. All cells were maintained at 37 °C with 5% CO_2_ in a humidified incubator and tested for mycoplasma monthly. For primary cell culture, female BALB/c and C57BL/6J mice (Charles River Laboratories, Sulzfeld, Germany) were euthanized with CO_2_. Spleen and lymph nodes (inguinal, axillar, and brachial) were dissociated using 40-µm cell strainers for T cell harvesting. Erythrocytes were removed with ACK lysis buffer (Lonza, Basel, Switzerland). CD4^+^ and CD8^+^ T cells were isolated using a MACS column system (Miltenyi; Bergisch Gladbach, Germany) and resuspended in DMEM containing 1% 1 M HEPES buffer (PAN-Biotech GmbH, Aidenbach, Germany), 1% 100 mM sodium pyruvate solution, 1% MEM amino acid solution, and 500 µM β-mercaptoethanol (all from Sigma Aldrich). The cells were cultured in 6-well plates prepared 24 h in advance with either phosphate-buffered saline (PBS) supplemented with 0.5 µg/mL anti-CD3 and 5 µg/mL anti-CD28 (both purchased from BioXcell, Lebanon, NH, USA) or exclusively with PBS (naïve condition). On the subsequent day, 50 U/mL IL-2 (Novartis, Basel, CH) and 0.5 ng/mL IL-7 (Thermo Scientific, Waltham, MA, USA) were added to the cells cultured with anti-CD3/anti-CD28 mAbs. After another 24 h, the cells were harvested and used for in vitro experiments.

For macrophage isolation, the femur and tibia of mice were flushed with PBS to collect the bone marrow. Erythrocytes were removed with ACK lysis buffer. To differentiate bone marrow-derived cells into macrophages, 20 ng/mL M-CSF (BioLegend, San Diego, CA, USA) was added to the culture medium (DMEM). After 6 days of culture, the macrophages were activated by the addition of fresh cell culture medium containing 10 ng/mL of lipopolysaccharide (LPS) (Sigma Aldrich), murine interferon-γ (IFN-γ) (Merck), murine tumor necrosis factor (TNF) (R&D Systems), or a combination of IFN-γ + TNF. The medium was also changed for untreated macrophages (M0), but no LPS, IFN-γ, or TNF was added. On day 7, the macrophages were washed with cold PBS, scraped from the flask, and used for further in vitro experiments.

The murine colon adenocarcinoma (MC38) and colorectal carcinoma (CT26) cell lines were a gift from M.F. Fransen (Department of Immunohematology and Blood Transfusion, Leiden University Medical Center (LUMC), The Netherlands); the mammary (4T1) cell line was kindly donated by M.A. Neveu (Laboratory of Tumor Inflammation and Angiogenesis, Department of Oncology, K.U. Leuven, Belgium). The murine melanoma (B16-F10) cell line was purchased from the American Type Culture Collection (ATCC, Rockville, MD, USA). MC38 and B16-F10 cells were maintained in DMEM (1% 1 M HEPES buffer was added to the culture media of MC38); 4T1 and CT26 cells were cultured in RPMI.

For in vivo experiments, 7-week-old female C57BL/6 J and BALB/c mice were briefly anesthetized with 1.5% isoflurane evaporated in oxygen (1.5 L/min) and inoculated in the right shoulder by subcutaneous (s.c.) or intracutaneous (i.c.) injection of 100,000 MC38 (s.c., C57BL/6J), 125,000 B16-F10 (i.c., C57BL/6 J), 100,000 CT26 (s.c., BALB/c), or 200,000 4T1 (s.c., BALB/c) cells in PBS. The animals were kept under standardized conditions with food and water ad libitum. The tumor volume (in mm^3^) was determined by measuring the length and width using a caliper and the following formula: V = (length*width*width)/2.

All animal experiments were performed in accordance with the German Animal Welfare Act and approved by the local authorities (Regierungspräsidium Tuebingen, Germany).

### Radiochemical synthesis of [^18^F]FAC

[^18^F]Fluoride was produced on a medical cyclotron (PETtrace 800, GE Healthcare, Uppsala, Sweden) using the ^18^O(p,n)^18^F nuclear reaction. [^18^F]FAC was produced as previously published^36,55^ on an ELIXYS FLEX/CHEM radiochemical synthesizer equipped with a PURE/FORM purification module (Sofie Biosciences, Dulles, VA, USA). The reference compound and precursor 1 (2-O-(trifluoromethylsulfonyl)-1,3,5-tri-O-benzoyl-alpha-D-ribo-furanose) were obtained from ABX (Radeberg, Germany). Precursor 2 (bis(trimethylsilyl)cytosine) was prepared by refluxing a solution of cytosine (0.5 g) and TMSCl (300 µL) in HMDS (10 mL) for 1 hour. The solvent was removed in vacuo at 50 °C and the resulting Precursor 2 was used without further purification^56^. Radiosynthesis yielded 4.0 ± 1.1 GBq [^18^F]FAC (6 ± 2% @EOS, RCP: 97 ± 4% (<0.3% fluorine-18), molar activity: 158 ± 87 GBq/µmol; n = 30).

### In vivo [^**18**^**F]FAC imaging** and data analysis

Imaging was performed via a 7 T small-animal MRI device (Bruker Biospin MRI; Germany) equipped with an in-house constructed and established MR-compatible PET insert^57,58^. The baseline PET and MR images were acquired 7–9 days after tumor cell inoculation, when the tumors reached a suitable size (50–70 mm^3^). A total of 7 ± 1 MBq [^18^F]FAC (50 µL) was injected intravenously (i.v.) per mouse, and after 60 min of resting uptake under inhalation anesthesia (1.5% isoflurane vaporized in oxygen; 1.5 L/min), a 10-min static PET scan was performed simultaneously with an anatomical MR scan using a T2-weighted 3-dimensional turbo spin-echo sequence. The body temperature of the anesthetized mice was maintained in the anesthesia chamber by a heating pad during the one-hour period of [^18^F]FAC uptake. Immediately after the baseline PET/MRI scan, the mice were intraperitoneally (i.p.) injected with either 200 µg of a PD-L1 blocking antibody (anti-mouse-PD-L1 mAb; clone 10 F.9G2; Bio X Cell, West Lebanon, NH, USA) or an IgG2b isotype antibody (control/sham treatment; clone LTF-2; Bio X Cell). The treatment was repeated every two to three days for a total period of one week (3 injections in total). On the second day after the last treatment (6–7 days after the onset of CIT), the mice underwent a follow-up PET/MRI scan as described above. The imaging schedule was chosen based on published studies demonstrating that immune activation in *TDLN* is an early event following CIT, typically detectable around days 3–5 and peaking 7–10 days after treatment initiation^13,14,59^. A similar early imaging window (24–48 h post onset of treatment) has been successfully applied using the related nucleoside tracer [¹⁸F]F-AraG^32^. Our study design was therefore optimized to capture the peak of T cell–driven immune activation in TDLNs, which represents a key hallmark of CIT–induced immune modulation. The acquired PET data were reconstructed with Inveon Acquisition Workplace (Siemens Preclinical Solutions, Knoxville, TN, USA) using Fourier rebinning and the OSEM 2D algorithm. Volumes of interest (VOIs) were created based on the MR images before they were fused to the respective PET scans using Inveon Research Workplace. The radioactivity fraction per tissue volume (percent injected dose per cubic centimeter; %ID/cc) was calculated based on the [^18^F]FAC uptake (Bq/mL) in the VOIs after correction for radioactive decay. After the follow-up PET/MRI scan, the mice were either processed for biodistribution or immediately euthanized with CO_2_ to collect their lymph nodes for ex vivo analysis of immune cell uptake of [^18^F]FAC or flow cytometry analyses.

### In vitro and ex vivo serum stability of [^18^F]FAC

The stability of [^18^F]FAC in murine serum was investigated through both in vitro and in vivo experiments. For in vitro evaluation, 60 µL of mouse (BALB/c) serum was incubated with 30 MBq of [^18^F]FAC at 37 °C. Samples were collected at various time points: 0, 30, 60, 120, and 240 min after the start of incubation. For the ex vivo assessment, two BALB/c mice were injected with 13 ± 1 MBq of [^18^F]FAC. The first mouse was sacrificed 5 min after the tracer injection, while the second mouse was sacrificed 60 min after the injection. Blood samples were collected from both groups of mice and subjected to centrifugation to obtain the serum. All the serum samples from both the in vitro and in vivo experiments were subsequently analyzed using thin-layer chromatography (stationary phase: silica gel 60 F_254_; mobile phase: acetone/water 90/10 v/v). The [^18^F]FAC quality control sample was used as a reference.

### Ex vivo biodistribution of [^18^F]FAC

Blood samples were collected retrobulbarily from the anesthetized animals, which were then euthanized with CO_2_. Organs were rapidly isolated and weighed, and the organ-specific radioactivity was measured with a gamma counter (Wizard 2; PerkinElmer, Waltham, MA, USA). After decay correction, the results are expressed as percentages of the injected dose of radioactivity per gram of tissue (%ID/g).

### Ex vivo immune cell uptake of [^18^F]FAC

T cells (CD4^+^ and CD8^+^) were isolated from TDLNs of anti-PD-L1 mAb-treated experimental mice via a MACS column system (Miltenyi, Bergisch Gladbach, Germany), and radioactivity was measured via a gamma counter. Uptake was quantified as the percentage of [^18^F]FAC per 10^6^ cells. Three lymph nodes were pooled for one isolation.

### In vitro [^18^F]FAC cell uptake assay

90 µL cell suspension containing 10^5^ tumor cells (MC38, CT26, B16-F10, and 4T1), differentially primed and activated macrophages (M0, M1 innate and M1 adaptive) or naïve as well as activated T cells (CD4^+^ and CD8^+^) was incubated in 96-well filter plates (MADVN6550, Merck Millipore, Darmstadt, Germany) with 60 µL of a 0.4 MBq/mL radiotracer solution. To assess nonspecific binding, the filter plates alone were incubated with the same [^18^F]FAC concentration under identical conditions. After 30, 60, 90, and 120 min of incubation at 37 °C, the cells were washed by vacuum filtration with medium (2 × 100 µL) through the plate. The filters containing the cells were individually transferred into tubes via a commercial punch kit (MAMP09608, Merck) and measured via a gamma counter. Additionally, 60 µL of the 0.4 MBq/mL radiotracer solution was measured separately via a gamma counter to quantify the maximum amount of added radioactivity/well (100% [^18^F]FAC). Cell uptake was quantified as the percentage of added radioactivity. The nonspecific binding of [^18^F]FAC was <0.1%.

### Histopathology and immunohistochemistry of the lymph nodes

Tumor-draining and contralateral lymph nodes from the animals used for biodistribution were fixed in 4% formalin immediately after isolation. For histology, 3–5 µm-thick sections were cut and stained with hematoxylin and eosin (H&E). IHC was performed on an automated immunostainer (Ventana Medical Systems, Inc., Oro Valley, AZ, USA) according to the company’s protocols for open procedures with slight modifications. All the samples were stained with primary antibodies against CD3 (Clone SP7; DCS Innovative Diagnostik-Systeme GmbH u. Co. KG, Hamburg, Germany) and B220 (Clone RA3-6B2; BD Biosciences, Becton, Dickinson and Company, Franklin Lakes, NJ, USA). Appropriate positive and negative controls were used to confirm the adequacy of the staining. All the samples were scanned with a Ventana DP200 (Roche, Basel, Switzerland) and processed with the Image Viewer MFC application. The final image was prepared using Adobe Photoshop CS6 (Adobe Inc., Mountain View, CA, USA). The images were analyzed via visual observation.

### Flow cytometry analysis

Single-cell suspensions were prepared from the draining and contralateral control lymph nodes. The cells were labeled ex vivo via the following fluorescent monoclonal antibodies: (FITC)-CD3ε (1:100; #152304; BioLegend), (PerCP)-CD4 (1:25; #100538; BioLegend), (AF700®)-CD8a (1:100; #100730; BioLegend), (BV421™)-CD25 (1:100; #102043; BioLegend), (BV650™)-CD69 (1:100; #104541; BioLegend), (BUV737)-CD11b (1:80; #612800; BD Bioscience), (BV711™)-MHCII (1:80; #107643; BioLegend), (PE/Dazzle™ 594)-F4/80 (1:80; #123146; BioLegend) and Zombie NIR™ (1:2000; #423105; BioLegend). The samples were analyzed using a Cytek® Aurora spectral flow cytometer (Cytek Biosciences B.V., Amsterdam, The Netherlands). The fluorescence data were analyzed using FlowJo (FlowJo, LLC, OR, USA) (Fig. S6).

### [^18^F]CFA-PET/CT scan

Patients with metastatic or recurrent advanced melanoma underwent [^18^F]CFA-PET/CT investigations within a noninterventional pilot study (NCT03409419) before and 2 to 4 weeks after immune therapy interventions (anti-PD1 mAb + anti-CTLA4 mAb). Eligible participants were adults (≥18 years old) with hematologic malignancies or solid tumors who were capable of complying with study procedures and able to remain still for the duration of the imaging session (up to one hour). All patients, or their legally acceptable representatives, provided written informed consent prior to study participation. Patients who were pregnant or nursing, had a creatinine clearance level of ≤60 mL/min, or had underlying diseases or other conditions that, in the judgment of the investigator, could interfere with the acquisition of complete and high-quality data were excluded.

A bolus of 130 MBq (3.5 mCi) [^18^F]CFA was administered intravenously; 60 min postinjection, patients were scanned using a PET/CT scanner (Biograph True Point 64; Siemens Medical Systems; low-dose ACCT, whole-body PET image acquisition: 3 min/bed position). PET images were reconstructed using corrections for attenuation, dead time, random events, and scatter. Lymph nodes were identified based on the corresponding CT images, regions of interest were manually delineated, and the maximum standardized uptake value (SUVmax) was determined.

### Statistical analysis

Statistical analyses were performed in GraphPad Prism 9 (GraphPad Software, Inc., San Diego, CA, USA). The values are presented as arithmetic means ± standard error mean (SEM). Statistical significance was calculated using a nonparametric Student’s *t*-test when two groups were compared or using two-way ANOVA plus Tukey’s multiple comparisons test when more than two groups were compared. Significance is indicated in the figures by *p-value < 0.05; **p-value < 0.01; ***p-value < 0.001; ****p-value < 0.0001.

## Supplementary information

Suppl. Material npjimaging resubmission

## Data Availability

The datasets generated and analyzed during the current study are available from the corresponding author upon reasonable request.
