# Supplementary material for: Noninvasive in vivo deoxycytidine kinase (dCK)-PET identifies tumor-draining lymph nodes upon immune checkpoint inhibitor therapy
Source: Npj Imaging. 2026 Jan 6;4:1. doi: 10.1038/s44303-025-00133-8 (PMC12775488; doi:10.1038/s44303-025-00133-8)
Supplement: Supplementary file 1 — Suppl. Material npjimaging resubmission [file 44303_2025_133_MOESM1_ESM.docx]

**Supplementary Material**

**
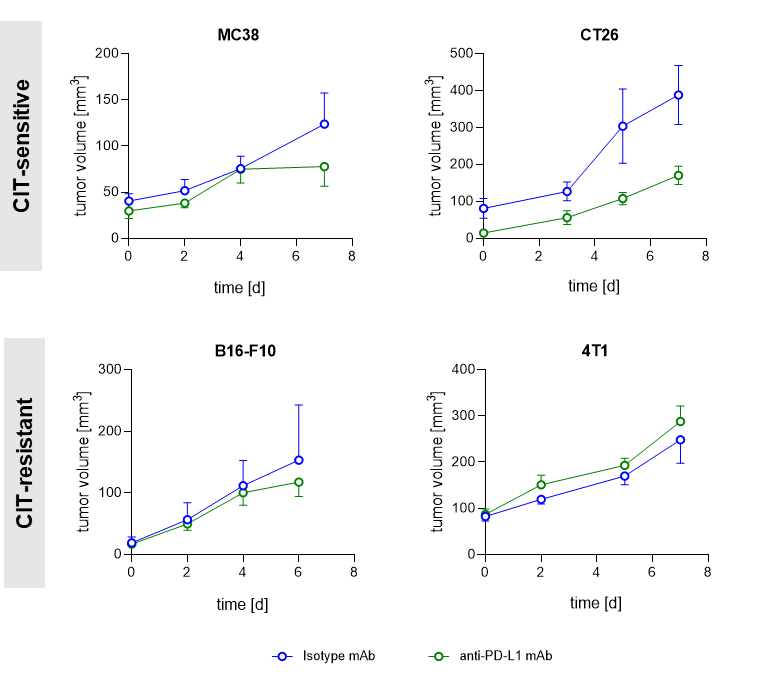
**

**

**Fig. S1.** Tumor growth over time during treatment with the anti-PD-L1 mAb or isotype control mAb (n=10–15 animals per group). The data are expressed as means ± SEM (**p<0.01).

**
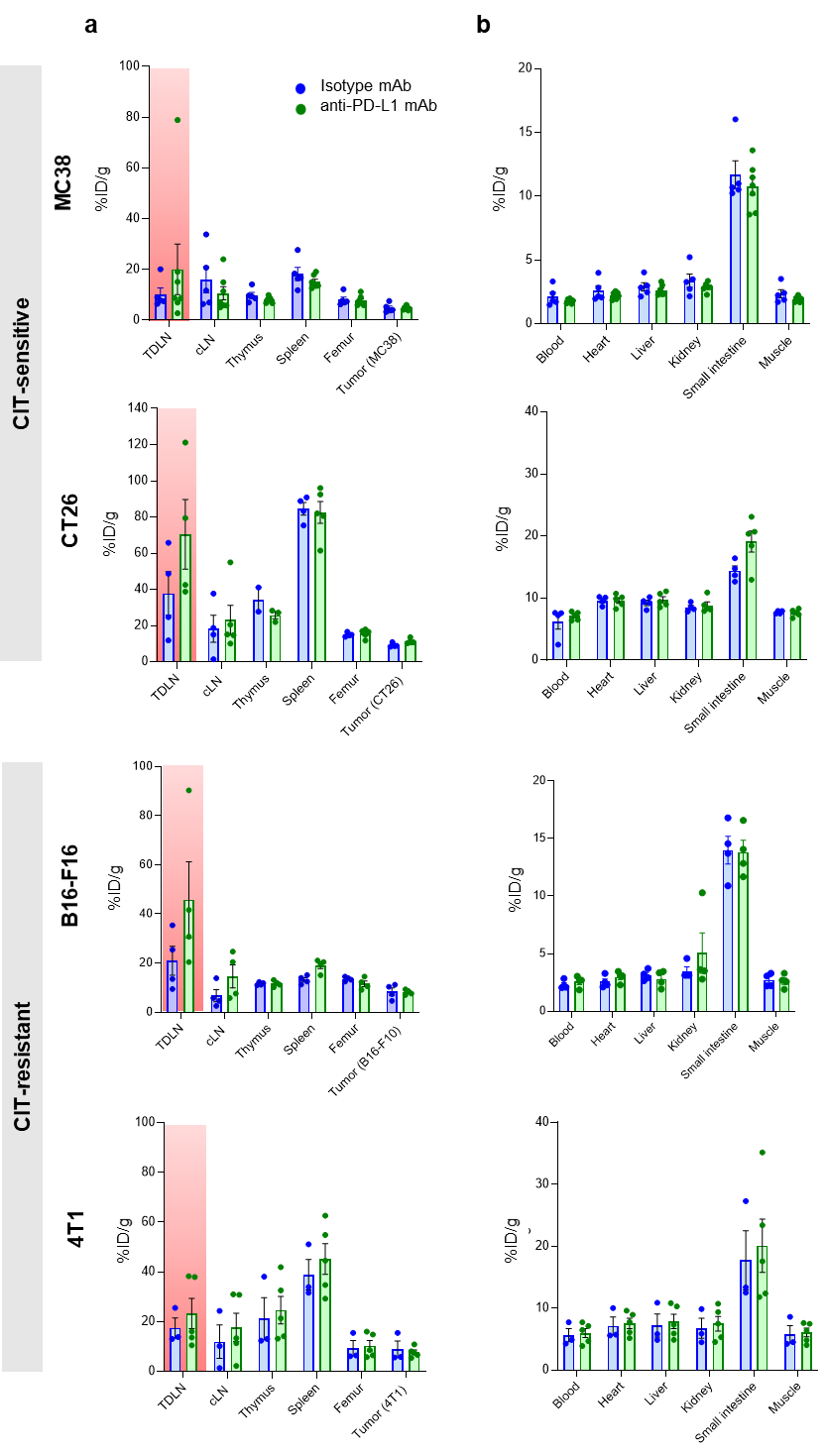
**

**Fig. S2.** (**a**) [^18^F]FAC ex vivo uptake quantification in primary and secondary lymphoid organs, as well as tumors. (**b**) [^18^F]FAC ex vivo uptake quantification in non-lymphoid organs (n = 3–7 animals per group). %ID/g = percentage injected dose per gram of tissue.


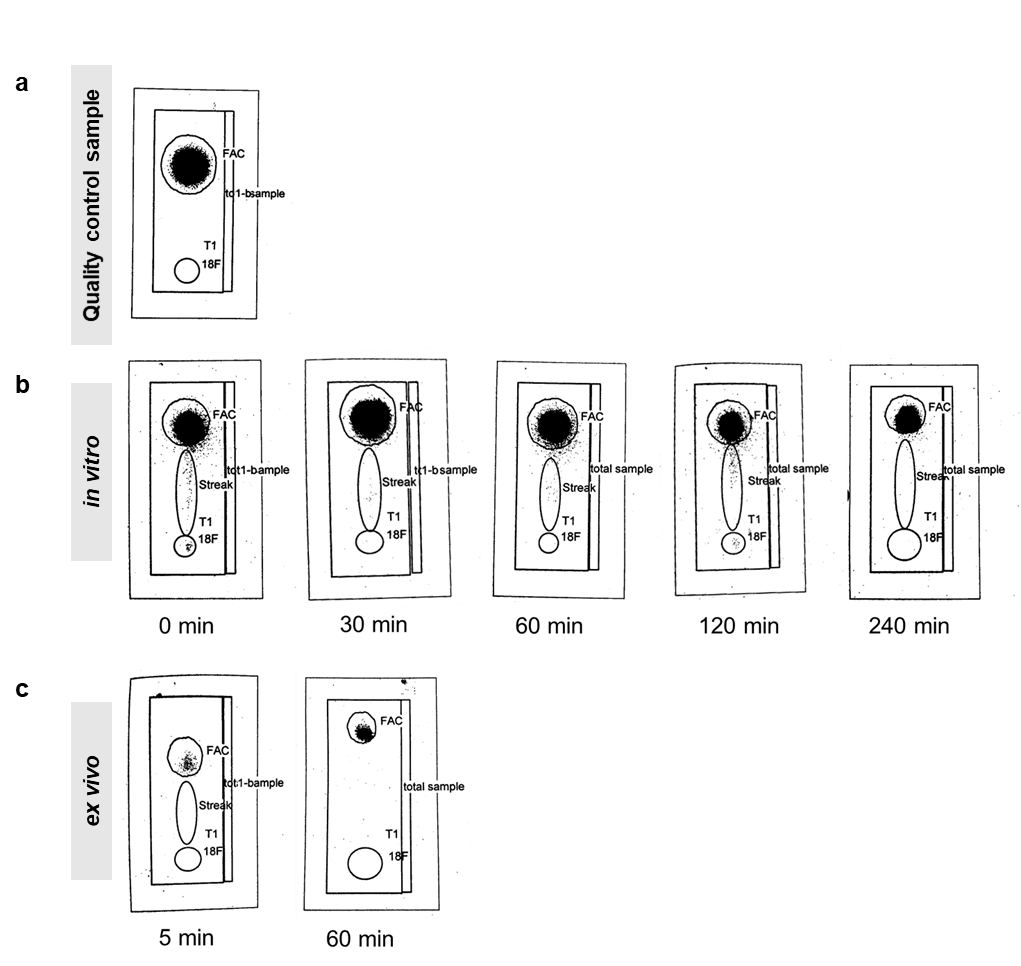


**Fig. S3.** Thin-layer chromatograms of [^18^F]FAC (**a**) quality control sample and samples from (**b**) in vitro and (**c**) ex vivo serum stability assessments.


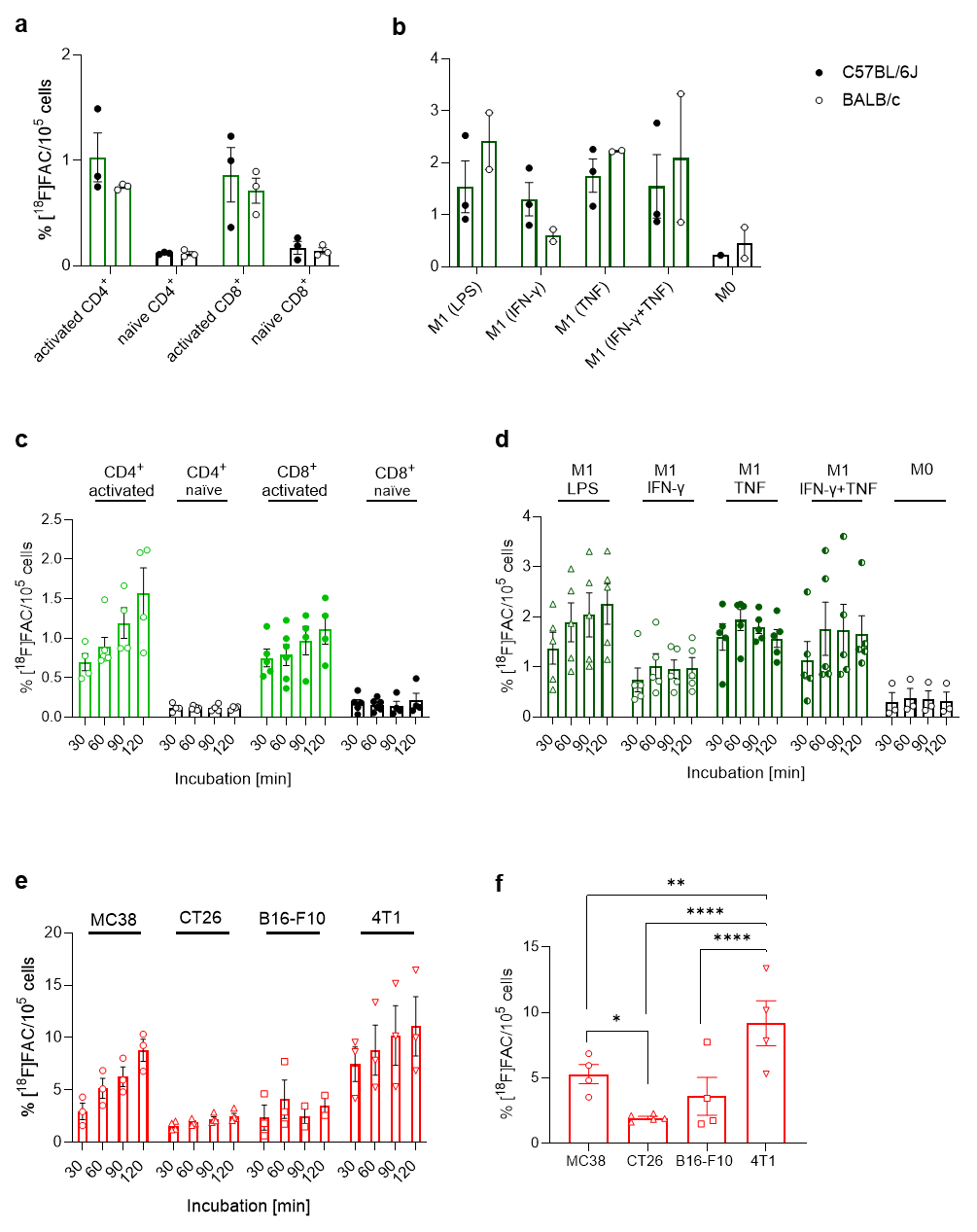


**Fig. S4**. In vitro (**a**) T cell and (**b**) macrophage uptake of [^18^F]FAC after 60 min of incubation. T cells and macrophages were isolated from either C57BL/6J or BALB/c mice. [^18^F]FAC uptake in (**c**) T cells and (**d**) macrophages over time (30–120 min). The uptake results from T cells and macrophages originating from C57BL/6J and BALB/c mice were combined. (**e**) Tumor cell uptake of [^18^F]FAC over time (30–120 min) and (**f**) after 60 min of incubation. The data are expressed as means ± SEM (**p*<0.05, ***p*<0.01, *****p*<0.0001) (n=4–5 individual experiments, each in duplicate).


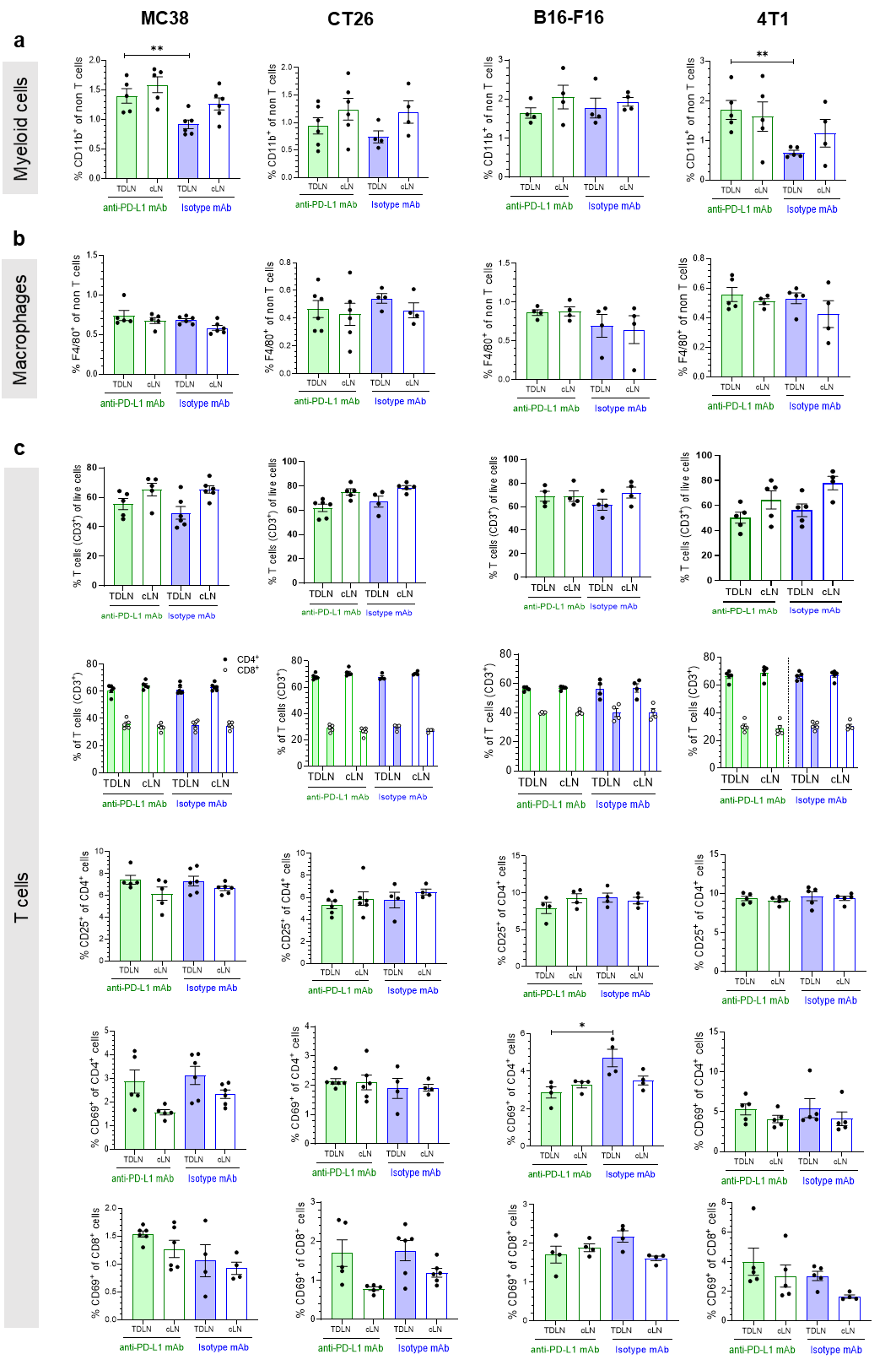


**Fig. S5.** Flow cytometry analyses of TDLNs and cLNs. Quantification of (**a**) myeloid cells (CD11b^+^), (**b**) macrophages (F4/80^+^) and (**c**) T cells (CD3^+^, CD4^+^, CD8^+^; activated T cells:CD4^+^CD25^+^, CD4^+^CD69^+^, CD8^+^CD69^+^), (n=4–6 LNs per group). The data are expressed as means ± SEM (**p*<0.05, ***p*<0.01). TDLN = tumor-draining lymph node, cLN = control (contralateral) lymph node.

**
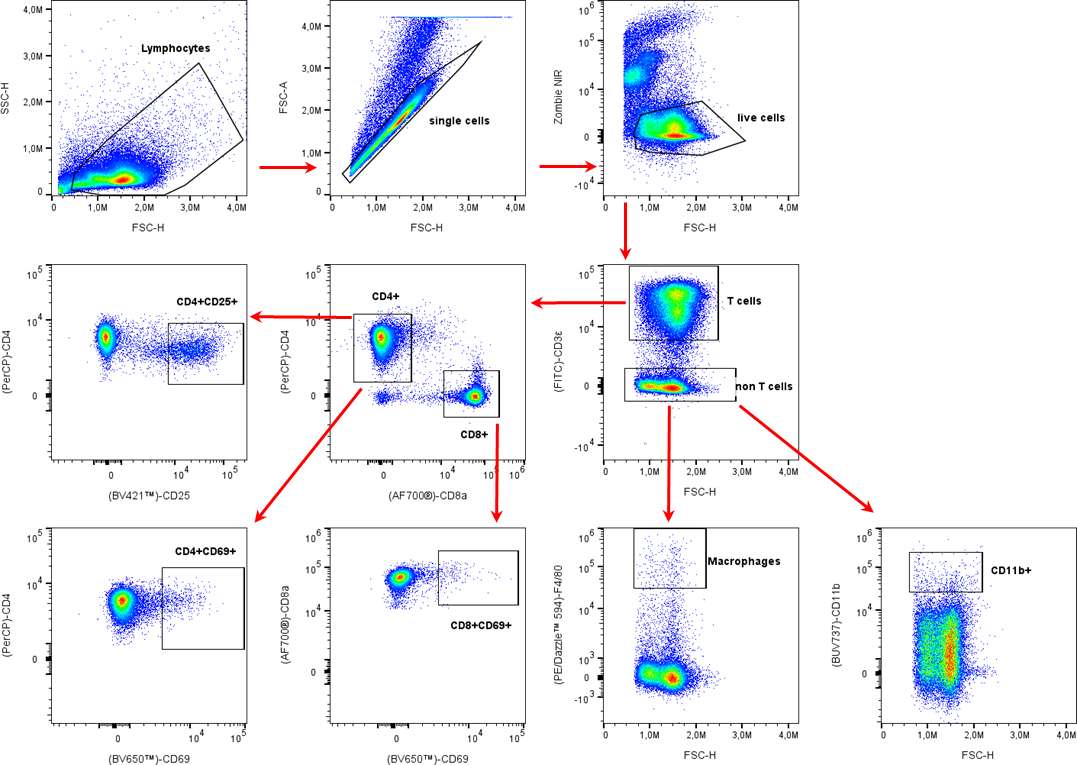
**

**Fig. S6.** Gating scheme of the flow cytometry data. Representative data from TDLNs of anti-PD-L1 mAb-treated MC38 tumor-bearing mice. The same gating scheme was used for all flow cytometry data.
